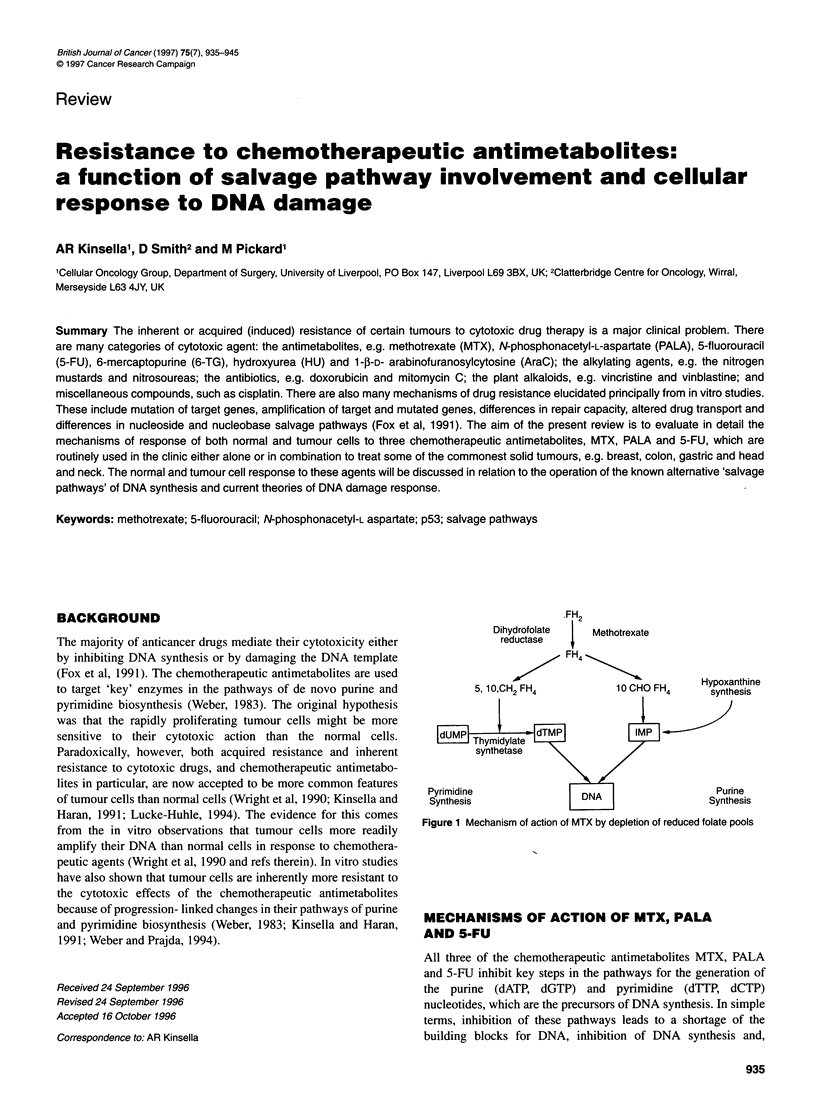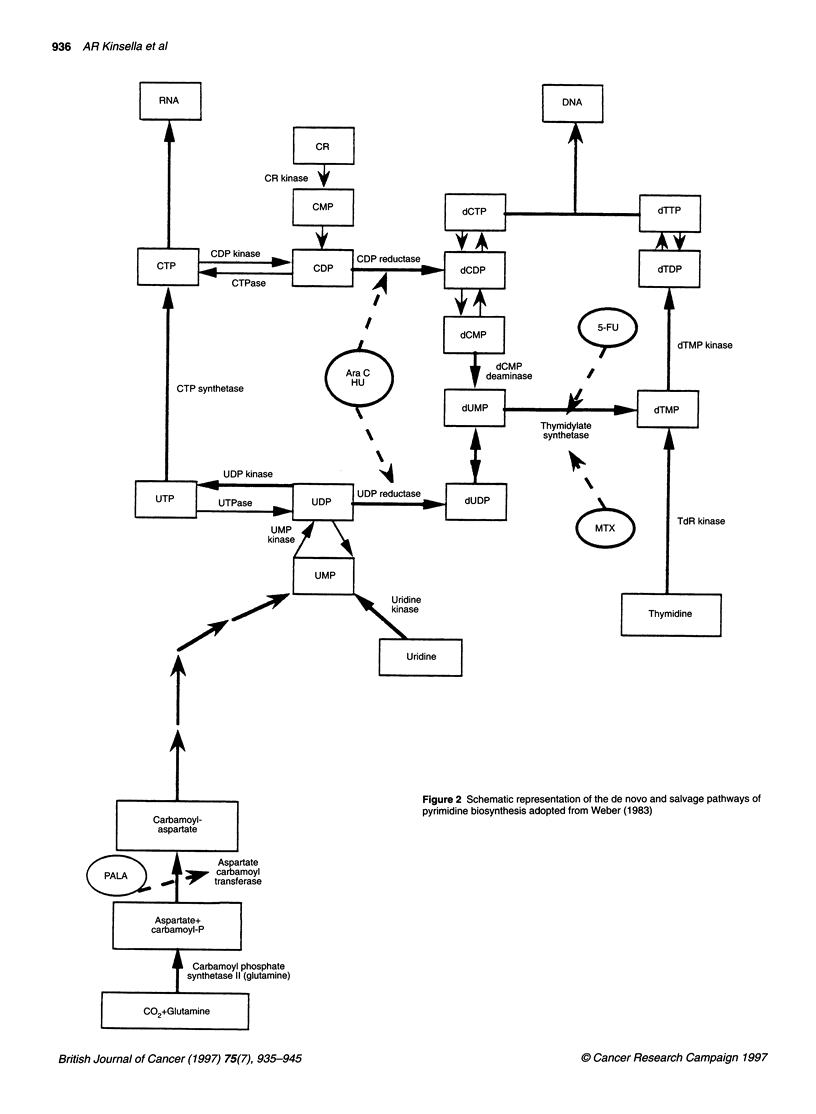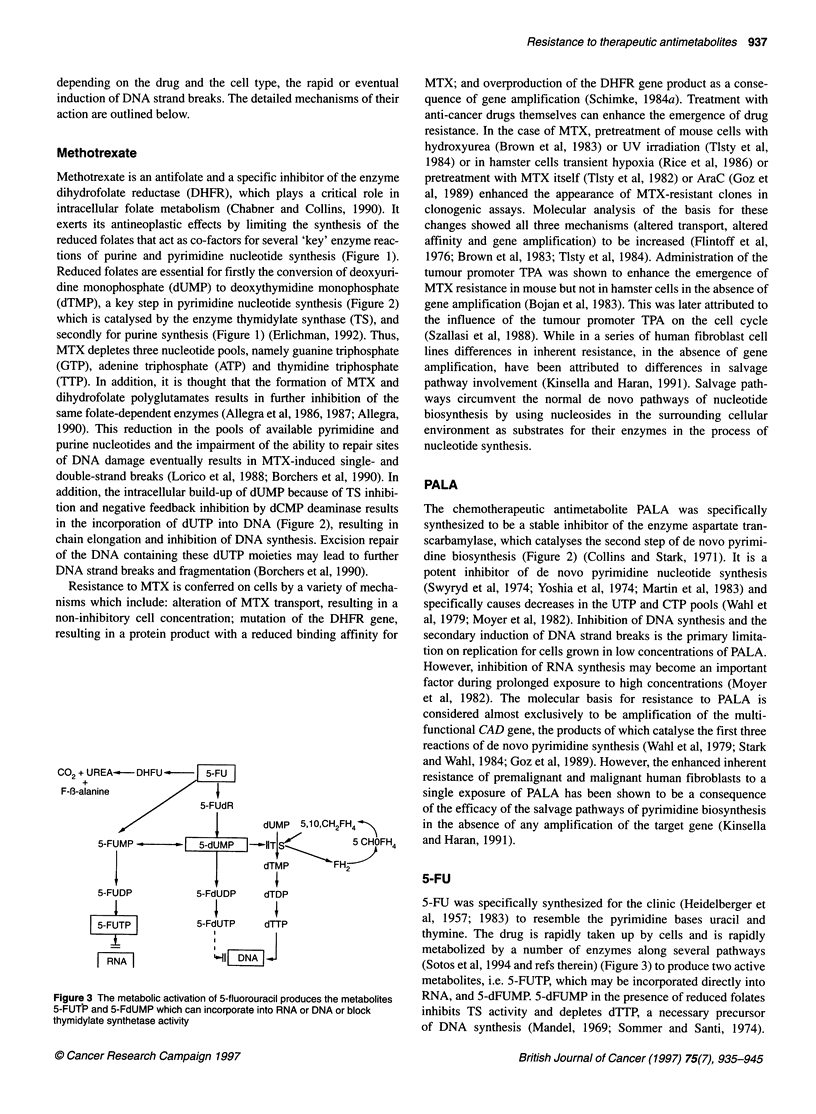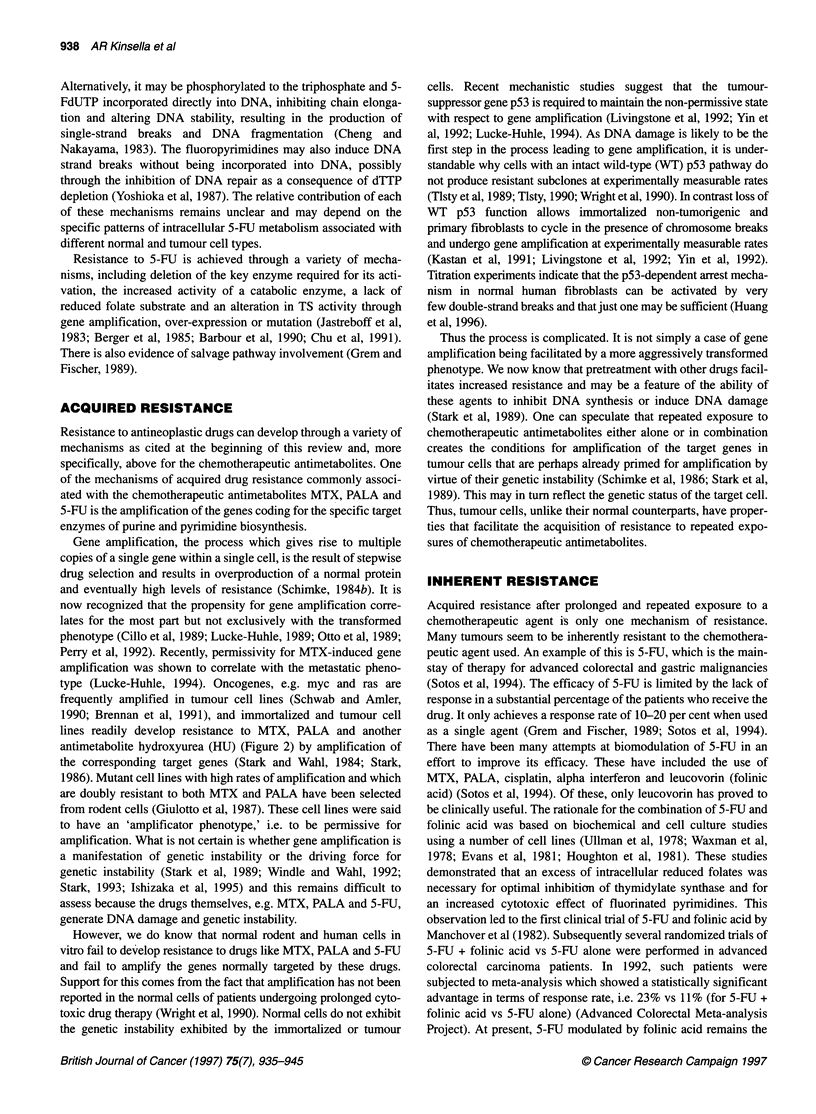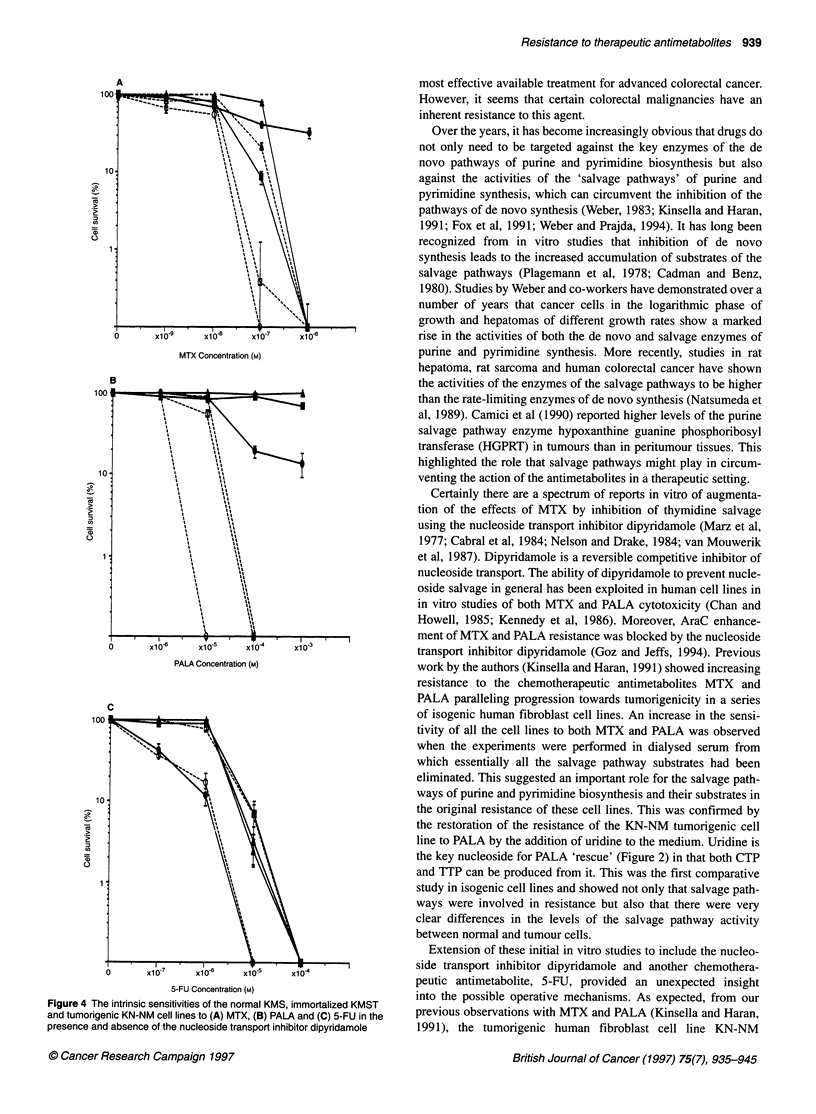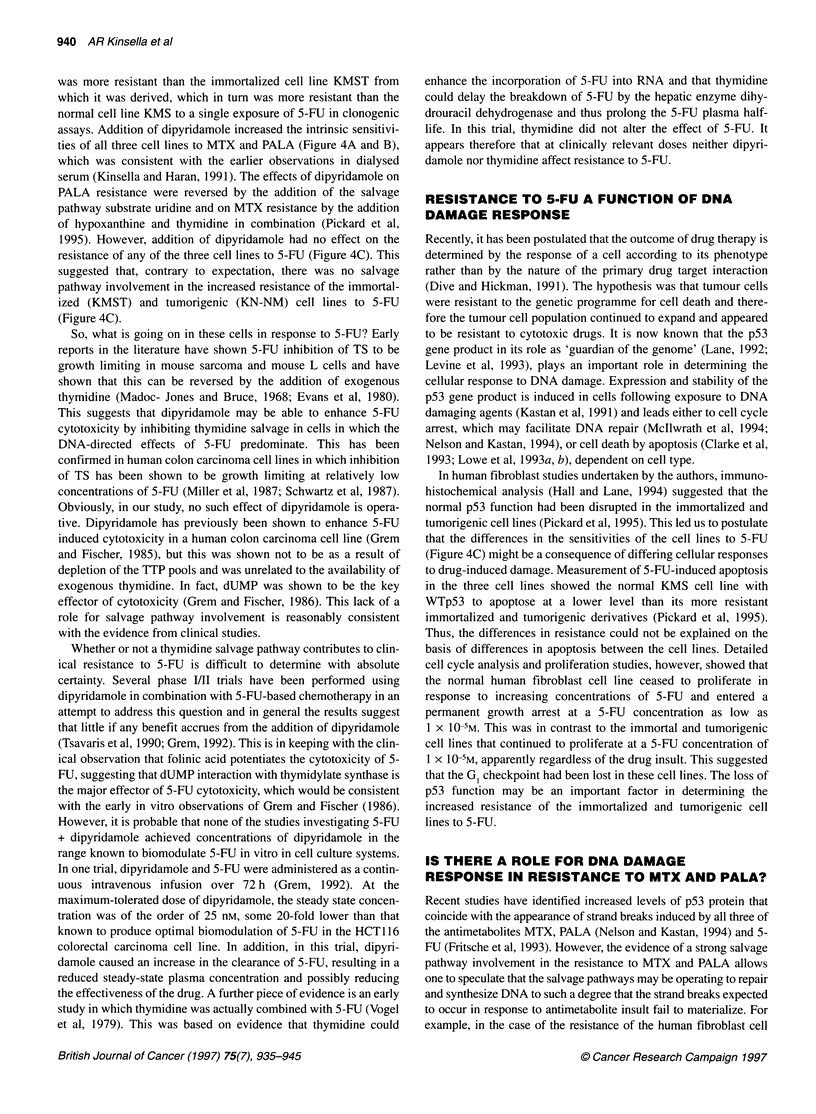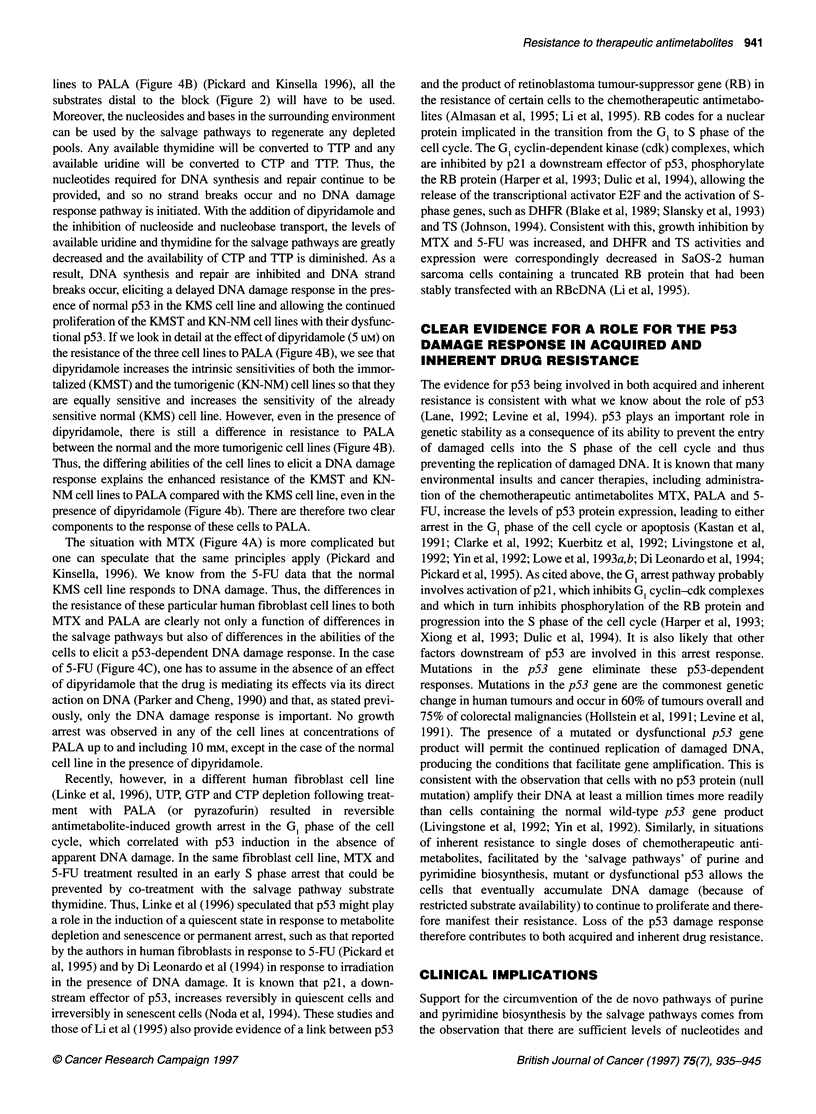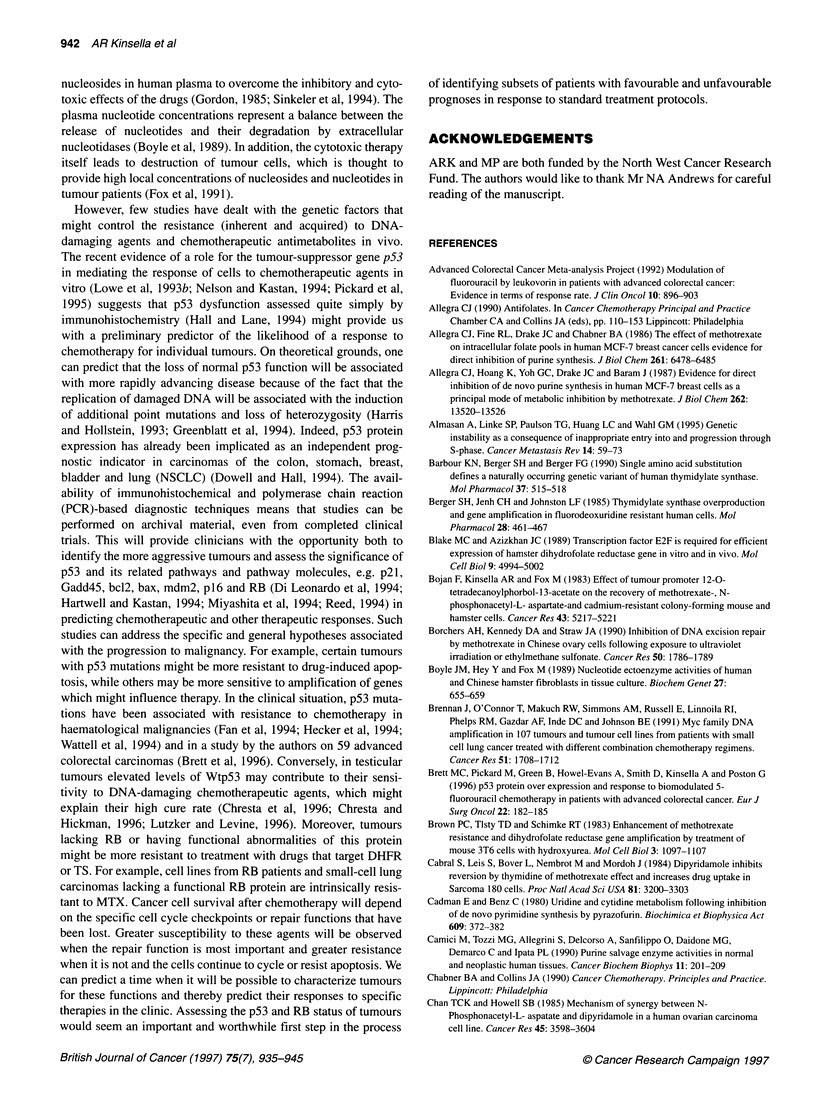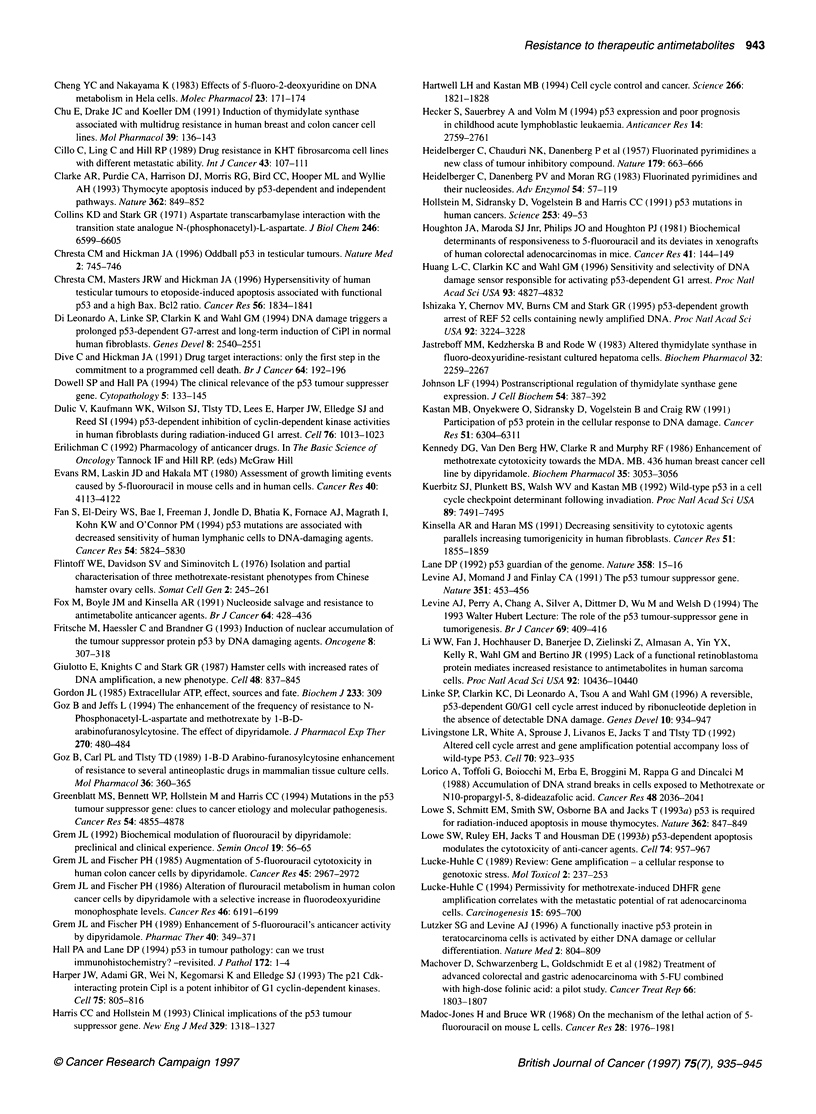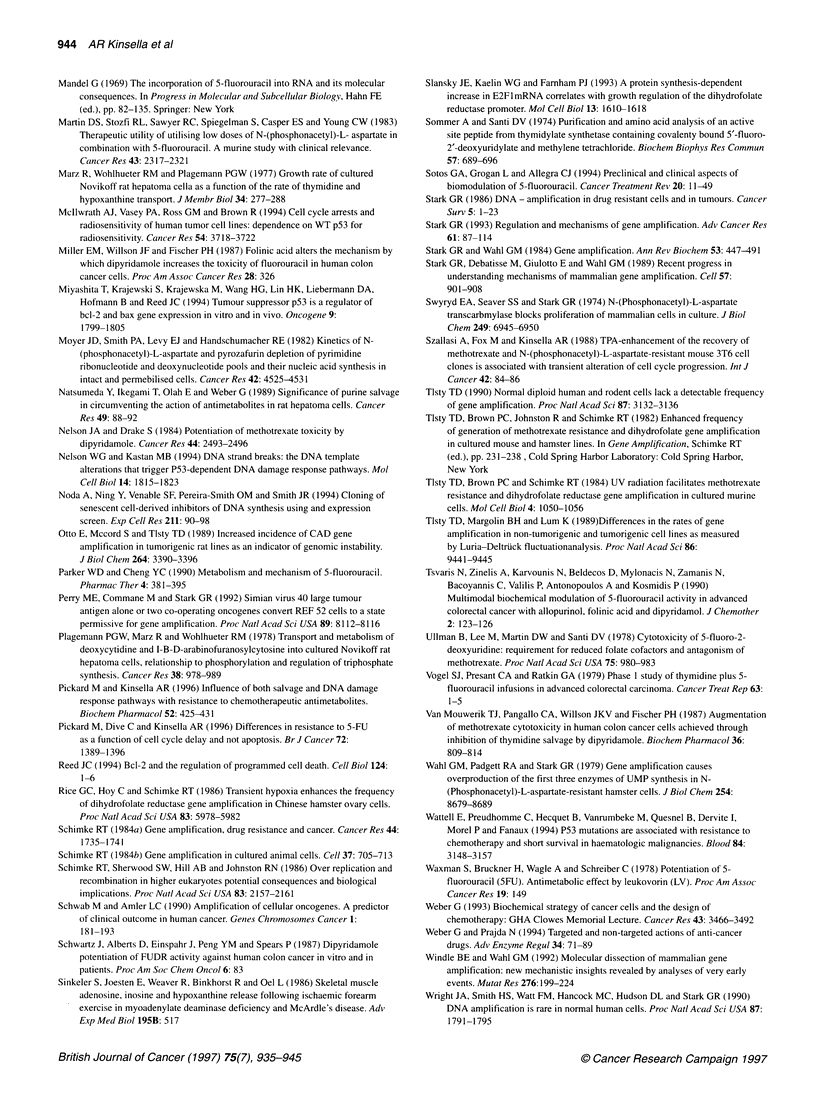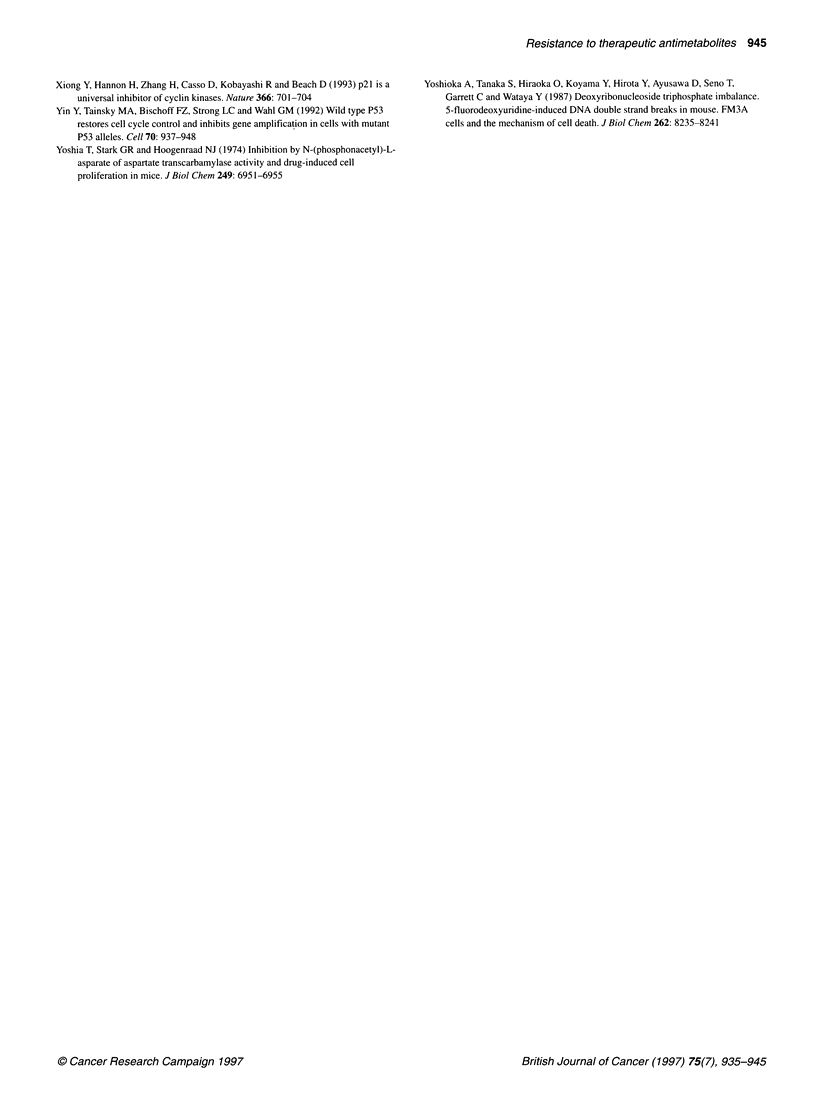# Resistance to chemotherapeutic antimetabolites: a function of salvage pathway involvement and cellular response to DNA damage.

**DOI:** 10.1038/bjc.1997.164

**Published:** 1997

**Authors:** A. R. Kinsella, D. Smith, M. Pickard

**Affiliations:** Department of Surgery, University of Liverpool, UK.

## Abstract

The inherent or acquired (induced) resistance of certain tumours to cytotoxic drug therapy is a major clinical problem. There are many categories of cytotoxic agent: the antimetabolites, e.g. methotrexate (MTX), N-phosphonacetyl-L-aspartate (PALA), 5-fluorouracil (5-FU), 6-mercaptopurine (6-TG), hydroxyurea (HU) and 1-beta-D-arabinofuranosylcytosine (AraC); the alkylating agents, e.g. the nitrogen mustards and nitrosoureas; the antibiotics, e.g. doxorubicin and mitomycin C; the plant alkaloids, e.g. vincristine and vinblastine; and miscellaneous compounds, such as cisplatin. There are also many mechanisms of drug resistance elucidated principally from in vitro studies. These include mutation of target genes, amplification of target and mutated genes, differences in repair capacity, altered drug transport and differences in nucleoside and nucleobase salvage pathways (Fox et al, 1991). The aim of the present review is to evaluate in detail the mechanisms of response of both normal and tumour cells to three chemotherapeutic antimetabolites, MTX, PALA and 5-FU, which are routinely used in the clinic either alone or in combination to treat some of the commonest solid tumours, e.g. breast, colon, gastric and head and neck. The normal and tumour cell response to these agents will be discussed in relation to the operation of the known alternative 'salvage pathways' of DNA synthesis and current theories of DNA damage response.


*British Journal of Cancer* (1997) **75**(7), 935–945
© 1997 Cancer Research Campaign

Review

### Resistance to chemotherapeutic antimetabolites: a function of salvage pathway involvement and cellular response to DNA damage

**AR Kinsella[1], D Smith[2] and M Pickard[1]**

[1]Cellular Oncology Group, Department of Surgery, University of Liverpool, PO Box 147, Liverpool L69 3BX, UK; [2]Clatterbridge Centre for Oncology, Wirral, Merseyside L63 4JY, UK

**Summary** The inherent or acquired (induced) resistance of certain tumours to cytotoxic drug therapy is a major clinical problem. There are many categories of cytotoxic agent: the antimetabolites, e.g. methotrexate (MTX), *N*-phosphonacetyl-L-aspartate (PALA), 5-fluorouracil (5-FU), 6-mercaptopurine (6-TG), hydroxyurea (HU) and 1-β-D- arabinofuranosylcytosine (AraC); the alkylating agents, e.g. the nitrogen mustards and nitrosoureas; the antibiotics, e.g. doxorubicin and mitomycin C; the plant alkaloids, e.g. vincristine and vinblastine; and miscellaneous compounds, such as cisplatin. There are also many mechanisms of drug resistance elucidated principally from in vitro studies. These include mutation of target genes, amplification of target and mutated genes, differences in repair capacity, altered drug transport and differences in nucleoside and nucleobase salvage pathways (Fox et al, 1991). The aim of the present review is to evaluate in detail the mechanisms of response of both normal and tumour cells to three chemotherapeutic antimetabolites, MTX, PALA and 5-FU, which are routinely used in the clinic either alone or in combination to treat some of the commonest solid tumours, e.g. breast, colon, gastric and head and neck. The normal and tumour cell response to these agents will be discussed in relation to the operation of the known alternative 'salvage pathways' of DNA synthesis and current theories of DNA damage response.

**Keywords:** methotrexate; 5-fluorouracil; *N*-phosphonacetyl-L aspartate; p53; salvage pathways

### BACKGROUND

The majority of anticancer drugs mediate their cytotoxicity either by inhibiting DNA synthesis or by damaging the DNA template (Fox et al, 1991). The chemotherapeutic antimetabolites are used to target 'key' enzymes in the pathways of de novo purine and pyrimidine biosynthesis (Weber, 1983). The original hypothesis was that the rapidly proliferating tumour cells might be more sensitive to their cytotoxic action than the normal cells. Paradoxically, however, both acquired resistance and inherent resistance to cytotoxic drugs, and chemotherapeutic antimetabolites in particular, are now accepted to be more common features of tumour cells than normal cells (Wright et al, 1990; Kinsella and Haran, 1991; Lucke-Huhle, 1994). The evidence for this comes from the in vitro observations that tumour cells more readily amplify their DNA than normal cells in response to chemotherapeutic agents (Wright et al, 1990 and refs therein). In vitro studies have also shown that tumour cells are inherently more resistant to the cytotoxic effects of the chemotherapeutic antimetabolites because of progression- linked changes in their pathways of purine and pyrimidine biosynthesis (Weber, 1983; Kinsella and Haran, 1991; Weber and Prajda, 1994).

*Received 24 September 1996*
*Revised 24 September 1996*
*Accepted 16 October 1996*

*Correspondence to:* AR Kinsella

**Figure 1** Mechanism of action of MTX by depletion of reduced folate pools

### MECHANISMS OF ACTION OF MTX, PALA AND 5-FU

All three of the chemotherapeutic antimetabolites MTX, PALA and 5-FU inhibit key steps in the pathways for the generation of the purine (dATP, dGTP) and pyrimidine (dTTP, dCTP) nucleotides, which are the precursors of DNA synthesis. In simple terms, inhibition of these pathways leads to a shortage of the building blocks for DNA, inhibition of DNA synthesis and,

**Figure 2** Schematic representation of the de novo and salvage pathways of pyrimidine biosynthesis adopted from Weber (1983)

*© Cancer Research Campaign 1997*

depending on the drug and the cell type, the rapid or eventual induction of DNA strand breaks. The detailed mechanisms of their action are outlined below.

### Methotrexate

Methotrexate is an antifolate and a specific inhibitor of the enzyme dihydrofolate reductase (DHFR), which plays a critical role in intracellular folate metabolism (Chabner and Collins, 1990). It exerts its antineoplastic effects by limiting the synthesis of the reduced folates that act as co-factors for several 'key' enzyme reactions of purine and pyrimidine nucleotide synthesis (Figure 1). Reduced folates are essential for firstly the conversion of deoxyuridine monophosphate (dUMP) to deoxythymidine monophosphate (dTMP), a key step in pyrimidine nucleotide synthesis (Figure 2) which is catalysed by the enzyme thymidylate synthase (TS), and secondly for purine synthesis (Figure 1) (Erlichman, 1992). Thus, MTX depletes three nucleotide pools, namely guanine triphosphate (GTP), adenine triphosphate (ATP) and thymidine triphosphate (TTP). In addition, it is thought that the formation of MTX and dihydrofolate polyglutamates results in further inhibition of the same folate-dependent enzymes (Allegra et al, 1986, 1987; Allegra, 1990). This reduction in the pools of available pyrimidine and purine nucleotides and the impairment of the ability to repair sites of DNA damage eventually results in MTX-induced single- and double-strand breaks (Lorico et al, 1988; Borchers et al, 1990). In addition, the intracellular build-up of dUMP because of TS inhibition and negative feedback inhibition by dCMP deaminase results in the incorporation of dUTP into DNA (Figure 2), resulting in chain elongation and inhibition of DNA synthesis. Excision repair of the DNA containing these dUTP moieties may lead to further DNA strand breaks and fragmentation (Borchers et al, 1990).

Resistance to MTX is conferred on cells by a variety of mechanisms which include: alteration of MTX transport, resulting in a non-inhibitory cell concentration; mutation of the DHFR gene, resulting in a protein product with a reduced binding affinity for MTX; and overproduction of the DHFR gene product as a consequence of gene amplification (Schimke, 1984*a*). Treatment with anti-cancer drugs themselves can enhance the emergence of drug resistance. In the case of MTX, pretreatment of mouse cells with hydroxyurea (Brown et al, 1983) or UV irradiation (Tlsty et al, 1984) or in hamster cells transient hypoxia (Rice et al, 1986) or pretreatment with MTX itself (Tlsty et al, 1982) or AraC (Goz et al, 1989) enhanced the appearance of MTX-resistant clones in clonogenic assays. Molecular analysis of the basis for these changes showed all three mechanisms (altered transport, altered affinity and gene amplification) to be increased (Flintoff et al, 1976; Brown et al, 1983; Tlsty et al, 1984). Administration of the tumour promoter TPA was shown to enhance the emergence of MTX resistance in mouse but not in hamster cells in the absence of gene amplification (Bojan et al, 1983). This was later attributed to the influence of the tumour promoter TPA on the cell cycle (Szallasi et al, 1988). While in a series of human fibroblast cell lines differences in inherent resistance, in the absence of gene amplification, have been attributed to differences in salvage pathway involvement (Kinsella and Haran, 1991). Salvage pathways circumvent the normal de novo pathways of nucleotide biosynthesis by using nucleosides in the surrounding cellular environment as substrates for their enzymes in the process of nucleotide synthesis.

### PALA

The chemotherapeutic antimetabolite PALA was specifically synthesized to be a stable inhibitor of the enzyme aspartate transcarbamylase, which catalyses the second step of de novo pyrimidine biosynthesis (Figure 2) (Collins and Stark, 1971). It is a potent inhibitor of de novo pyrimidine nucleotide synthesis (Swyryd et al, 1974; Yoshia et al, 1974; Martin et al, 1983) and specifically causes decreases in the UTP and CTP pools (Wahl et al, 1979; Moyer et al, 1982). Inhibition of DNA synthesis and the secondary induction of DNA strand breaks is the primary limitation on replication for cells grown in low concentrations of PALA. However, inhibition of RNA synthesis may become an important factor during prolonged exposure to high concentrations (Moyer et al, 1982). The molecular basis for resistance to PALA is considered almost exclusively to be amplification of the multifunctional *CAD* gene, the products of which catalyse the first three reactions of de novo pyrimidine synthesis (Wahl et al, 1979; Stark and Wahl, 1984; Goz et al, 1989). However, the enhanced inherent resistance of premalignant and malignant human fibroblasts to a single exposure of PALA has been shown to be a consequence of the efficacy of the salvage pathways of pyrimidine biosynthesis in the absence of any amplification of the target gene (Kinsella and Haran, 1991).

**Figure 3** The metabolic activation of 5-fluorouracil produces the metabolites 5-FUTP and 5-FdUMP which can incorporate into RNA or DNA or block thymidylate synthetase activity

### 5-FU

5-FU was specifically synthesized for the clinic (Heidelberger et al, 1957; 1983) to resemble the pyrimidine bases uracil and thymine. The drug is rapidly taken up by cells and is rapidly metabolized by a number of enzymes along several pathways (Sotos et al, 1994 and refs therein) (Figure 3) to produce two active metabolites, i.e. 5-FUTP, which may be incorporated directly into RNA, and 5-dFUMP. 5-dFUMP in the presence of reduced folates inhibits TS activity and depletes dTTP, a necessary precursor of DNA synthesis (Mandel, 1969; Sommer and Santi, 1974).

© Cancer Research Campaign 1997

Alternatively, it may be phosphorylated to the triphosphate and 5-FdUTP incorporated directly into DNA, inhibiting chain elongation and altering DNA stability, resulting in the production of single-strand breaks and DNA fragmentation (Cheng and Nakayama, 1983). The fluoropyrimidines may also induce DNA strand breaks without being incorporated into DNA, possibly through the inhibition of DNA repair as a consequence of dTTP depletion (Yoshioka et al, 1987). The relative contribution of each of these mechanisms remains unclear and may depend on the specific patterns of intracellular 5-FU metabolism associated with different normal and tumour cell types.

Resistance to 5-FU is achieved through a variety of mechanisms, including deletion of the key enzyme required for its activation, the increased activity of a catabolic enzyme, a lack of reduced folate substrate and an alteration in TS activity through gene amplification, over-expression or mutation (Jastreboff et al, 1983; Berger et al, 1985; Barbour et al, 1990; Chu et al, 1991). There is also evidence of salvage pathway involvement (Grem and Fischer, 1989).

### ACQUIRED RESISTANCE

Resistance to antineoplastic drugs can develop through a variety of mechanisms as cited at the beginning of this review and, more specifically, above for the chemotherapeutic antimetabolites. One of the mechanisms of acquired drug resistance commonly associated with the chemotherapeutic antimetabolites MTX, PALA and 5-FU is the amplification of the genes coding for the specific target enzymes of purine and pyrimidine biosynthesis.

Gene amplification, the process which gives rise to multiple copies of a single gene within a single cell, is the result of stepwise drug selection and results in overproduction of a normal protein and eventually high levels of resistance (Schimke, 1984*b*). It is now recognized that the propensity for gene amplification correlates for the most part but not exclusively with the transformed phenotype (Cillo et al, 1989; Lucke-Huhle, 1989; Otto et al, 1989; Perry et al, 1992). Recently, permissivity for MTX-induced gene amplification was shown to correlate with the metastatic phenotype (Lucke-Huhle, 1994). Oncogenes, e.g. myc and ras are frequently amplified in tumour cell lines (Schwab and Amler, 1990; Brennan et al, 1991), and immortalized and tumour cell lines readily develop resistance to MTX, PALA and another antimetabolite hydroxyurea (HU) (Figure 2) by amplification of the corresponding target genes (Stark and Wahl, 1984; Stark, 1986). Mutant cell lines with high rates of amplification and which are doubly resistant to both MTX and PALA have been selected from rodent cells (Giulotto et al, 1987). These cell lines were said to have an 'amplificator phenotype,' i.e. to be permissive for amplification. What is not certain is whether gene amplification is a manifestation of genetic instability or the driving force for genetic instability (Stark et al, 1989; Windle and Wahl, 1992; Stark, 1993; Ishizaka et al, 1995) and this remains difficult to assess because the drugs themselves, e.g. MTX, PALA and 5-FU, generate DNA damage and genetic instability.

However, we do know that normal rodent and human cells in vitro fail to develop resistance to drugs like MTX, PALA and 5-FU and fail to amplify the genes normally targeted by these drugs. Support for this comes from the fact that amplification has not been reported in the normal cells of patients undergoing prolonged cytotoxic drug therapy (Wright et al, 1990). Normal cells do not exhibit the genetic instability exhibited by the immortalized or tumour cells. Recent mechanistic studies suggest that the tumour-suppressor gene p53 is required to maintain the non-permissive state with respect to gene amplification (Livingstone et al, 1992; Yin et al, 1992; Lucke-Huhle, 1994). As DNA damage is likely to be the first step in the process leading to gene amplification, it is understandable why cells with an intact wild-type (WT) p53 pathway do not produce resistant subclones at experimentally measurable rates (Tlsty et al, 1989; Tlsty, 1990; Wright et al, 1990). In contrast loss of WT p53 function allows immortalized non-tumorigenic and primary fibroblasts to cycle in the presence of chromosome breaks and undergo gene amplification at experimentally measurable rates (Kastan et al, 1991; Livingstone et al, 1992; Yin et al, 1992). Titration experiments indicate that the p53-dependent arrest mechanism in normal human fibroblasts can be activated by very few double-strand breaks and that just one may be sufficient (Huang et al, 1996).

Thus the process is complicated. It is not simply a case of gene amplification being facilitated by a more aggressively transformed phenotype. We now know that pretreatment with other drugs facilitates increased resistance and may be a feature of the ability of these agents to inhibit DNA synthesis or induce DNA damage (Stark et al, 1989). One can speculate that repeated exposure to chemotherapeutic antimetabolites either alone or in combination creates the conditions for amplification of the target genes in tumour cells that are perhaps already primed for amplification by virtue of their genetic instability (Schimke et al, 1986; Stark et al, 1989). This may in turn reflect the genetic status of the target cell. Thus, tumour cells, unlike their normal counterparts, have properties that facilitate the acquisition of resistance to repeated exposures of chemotherapeutic antimetabolites.

### INHERENT RESISTANCE

Acquired resistance after prolonged and repeated exposure to a chemotherapeutic agent is only one mechanism of resistance. Many tumours seem to be inherently resistant to the chemotherapeutic agent used. An example of this is 5-FU, which is the mainstay of therapy for advanced colorectal and gastric malignancies (Sotos et al, 1994). The efficacy of 5-FU is limited by the lack of response in a substantial percentage of the patients who receive the drug. It only achieves a response rate of 10–20 per cent when used as a single agent (Grem and Fischer, 1989; Sotos et al, 1994). There have been many attempts at biomodulation of 5-FU in an effort to improve its efficacy. These have included the use of MTX, PALA, cisplatin, alpha interferon and leucovorin (folinic acid) (Sotos et al, 1994). Of these, only leucovorin has proved to be clinically useful. The rationale for the combination of 5-FU and folinic acid was based on biochemical and cell culture studies using a number of cell lines (Ullman et al, 1978; Waxman et al, 1978; Evans et al, 1981; Houghton et al, 1981). These studies demonstrated that an excess of intracellular reduced folates was necessary for optimal inhibition of thymidylate synthase and for an increased cytotoxic effect of fluorinated pyrimidines. This observation led to the first clinical trial of 5-FU and folinic acid by Manchover et al (1982). Subsequently several randomized trials of 5-FU + folinic acid vs 5-FU alone were performed in advanced colorectal carcinoma patients. In 1992, such patients were subjected to meta-analysis which showed a statistically significant advantage in terms of response rate, i.e. 23% vs 11% (for 5-FU + folinic acid vs 5-FU alone) (Advanced Colorectal Meta-analysis Project). At present, 5-FU modulated by folinic acid remains the

© Cancer Research Campaign 1997

**Figure 4** The intrinsic sensitivities of the normal KMS, immortalized KMST and tumorigenic KN-NM cell lines to (**A**) MTX, (**B**) PALA and (**C**) 5-FU in the presence and absence of the nucleoside transport inhibitor dipyridamole most effective available treatment for advanced colorectal cancer. However, it seems that certain colorectal malignancies have an inherent resistance to this agent.

Over the years, it has become increasingly obvious that drugs do not only need to be targeted against the key enzymes of the de novo pathways of purine and pyrimidine biosynthesis but also against the activities of the 'salvage pathways' of purine and pyrimidine synthesis, which can circumvent the inhibition of the pathways of de novo synthesis (Weber, 1983; Kinsella and Haran, 1991; Fox et al, 1991; Weber and Prajda, 1994). It has long been recognized from in vitro studies that inhibition of de novo synthesis leads to the increased accumulation of substrates of the salvage pathways (Plagemann et al, 1978; Cadman and Benz, 1980). Studies by Weber and co-workers have demonstrated over a number of years that cancer cells in the logarithmic phase of growth and hepatomas of different growth rates show a marked rise in the activities of both the de novo and salvage enzymes of purine and pyrimidine synthesis. More recently, studies in rat hepatoma, rat sarcoma and human colorectal cancer have shown the activities of the enzymes of the salvage pathways to be higher than the rate-limiting enzymes of de novo synthesis (Natsumeda et al, 1989). Camici et al (1990) reported higher levels of the purine salvage pathway enzyme hypoxanthine guanine phosphoribosyl transferase (HGPRT) in tumours than in peritumour tissues. This highlighted the role that salvage pathways might play in circumventing the action of the antimetabolites in a therapeutic setting.

Certainly there are a spectrum of reports in vitro of augmentation of the effects of MTX by inhibition of thymidine salvage using the nucleoside transport inhibitor dipyridamole (Marz et al, 1977; Cabral et al, 1984; Nelson and Drake, 1984; van Mouwerik et al, 1987). Dipyridamole is a reversible competitive inhibitor of nucleoside transport. The ability of dipyridamole to prevent nucleoside salvage in general has been exploited in human cell lines in in vitro studies of both MTX and PALA cytotoxicity (Chan and Howell, 1985; Kennedy et al, 1986). Moreover, AraC enhancement of MTX and PALA resistance was blocked by the nucleoside transport inhibitor dipyridamole (Goz and Jeffs, 1994). Previous work by the authors (Kinsella and Haran, 1991) showed increasing resistance to the chemotherapeutic antimetabolites MTX and PALA paralleling progression towards tumorigenicity in a series of isogenic human fibroblast cell lines. An increase in the sensitivity of all the cell lines to both MTX and PALA was observed when the experiments were performed in dialysed serum from which essentially all the salvage pathway substrates had been eliminated. This suggested an important role for the salvage pathways of purine and pyrimidine biosynthesis and their substrates in the original resistance of these cell lines. This was confirmed by the restoration of the resistance of the KN-NM tumorigenic cell line to PALA by the addition of uridine to the medium. Uridine is the key nucleoside for PALA 'rescue' (Figure 2) in that both CTP and TTP can be produced from it. This was the first comparative study in isogenic cell lines and showed not only that salvage pathways were involved in resistance but also that there were very clear differences in the levels of the salvage pathway activity between normal and tumour cells.

Extension of these initial in vitro studies to include the nucleoside transport inhibitor dipyridamole and another chemotherapeutic antimetabolite, 5-FU, provided an unexpected insight into the possible operative mechanisms. As expected, from our previous observations with MTX and PALA (Kinsella and Haran, 1991), the tumorigenic human fibroblast cell line KN-NM

© Cancer Research Campaign 1997

was more resistant than the immortalized cell line KMST from which it was derived, which in turn was more resistant than the normal cell line KMS to a single exposure of 5-FU in clonogenic assays. Addition of dipyridamole increased the intrinsic sensitivities of all three cell lines to MTX and PALA (Figure 4A and B), which was consistent with the earlier observations in dialysed serum (Kinsella and Haran, 1991). The effects of dipyridamole on PALA resistance were reversed by the addition of the salvage pathway substrate uridine and on MTX resistance by the addition of hypoxanthine and thymidine in combination (Pickard et al, 1995). However, addition of dipyridamole had no effect on the resistance of any of the three cell lines to 5-FU (Figure 4C). This suggested that, contrary to expectation, there was no salvage pathway involvement in the increased resistance of the immortalized (KMST) and tumorigenic (KN-NM) cell lines to 5-FU (Figure 4C).

So, what is going on in these cells in response to 5-FU? Early reports in the literature have shown 5-FU inhibition of TS to be growth limiting in mouse sarcoma and mouse L cells and have shown that this can be reversed by the addition of exogenous thymidine (Madoc- Jones and Bruce, 1968; Evans et al, 1980). This suggests that dipyridamole may be able to enhance 5-FU cytotoxicity by inhibiting thymidine salvage in cells in which the DNA-directed effects of 5-FU predominate. This has been confirmed in human colon carcinoma cell lines in which inhibition of TS has been shown to be growth limiting at relatively low concentrations of 5-FU (Miller et al, 1987; Schwartz et al, 1987). Obviously, in our study, no such effect of dipyridamole is operative. Dipyridamole has previously been shown to enhance 5-FU induced cytotoxicity in a human colon carcinoma cell line (Grem and Fischer, 1985), but this was shown not to be as a result of depletion of the TTP pools and was unrelated to the availability of exogenous thymidine. In fact, dUMP was shown to be the key effector of cytotoxicity (Grem and Fischer, 1986). This lack of a role for salvage pathway involvement is reasonably consistent with the evidence from clinical studies.

Whether or not a thymidine salvage pathway contributes to clinical resistance to 5-FU is difficult to determine with absolute certainty. Several phase I/II trials have been performed using dipyridamole in combination with 5-FU-based chemotherapy in an attempt to address this question and in general the results suggest that little if any benefit accrues from the addition of dipyridamole (Tsavaris et al, 1990; Grem, 1992). This is in keeping with the clinical observation that folinic acid potentiates the cytotoxicity of 5-FU, suggesting that dUMP interaction with thymidylate synthase is the major effector of 5-FU cytotoxicity, which would be consistent with the early in vitro observations of Grem and Fischer (1986). However, it is probable that none of the studies investigating 5-FU + dipyridamole achieved concentrations of dipyridamole in the range known to biomodulate 5-FU in vitro in cell culture systems. In one trial, dipyridamole and 5-FU were administered as a continuous intravenous infusion over 72 h (Grem, 1992). At the maximum-tolerated dose of dipyridamole, the steady state concentration was of the order of 25 nM, some 20-fold lower than that known to produce optimal biomodulation of 5-FU in the HCT116 colorectal carcinoma cell line. In addition, in this trial, dipyridamole caused an increase in the clearance of 5-FU, resulting in a reduced steady-state plasma concentration and possibly reducing the effectiveness of the drug. A further piece of evidence is an early study in which thymidine was actually combined with 5-FU (Vogel et al, 1979). This was based on evidence that thymidine could enhance the incorporation of 5-FU into RNA and that thymidine could delay the breakdown of 5-FU by the hepatic enzyme dihydrouracil dehydrogenase and thus prolong the 5-FU plasma half-life. In this trial, thymidine did not alter the effect of 5-FU. It appears therefore that at clinically relevant doses neither dipyridamole nor thymidine affect resistance to 5-FU.

### RESISTANCE TO 5-FU A FUNCTION OF DNA DAMAGE RESPONSE

Recently, it has been postulated that the outcome of drug therapy is determined by the response of a cell according to its phenotype rather than by the nature of the primary drug target interaction (Dive and Hickman, 1991). The hypothesis was that tumour cells were resistant to the genetic programme for cell death and therefore the tumour cell population continued to expand and appeared to be resistant to cytotoxic drugs. It is now known that the p53 gene product in its role as 'guardian of the genome' (Lane, 1992; Levine et al, 1993), plays an important role in determining the cellular response to DNA damage. Expression and stability of the p53 gene product is induced in cells following exposure to DNA damaging agents (Kastan et al, 1991) and leads either to cell cycle arrest, which may facilitate DNA repair (McIlwrath et al, 1994; Nelson and Kastan, 1994), or cell death by apoptosis (Clarke et al, 1993; Lowe et al, 1993*a*, *b*), dependent on cell type.

In human fibroblast studies undertaken by the authors, immunohistochemical analysis (Hall and Lane, 1994) suggested that the normal p53 function had been disrupted in the immortalized and tumorigenic cell lines (Pickard et al, 1995). This led us to postulate that the differences in the sensitivities of the cell lines to 5-FU (Figure 4C) might be a consequence of differing cellular responses to drug-induced damage. Measurement of 5-FU-induced apoptosis in the three cell lines showed the normal KMS cell line with WTp53 to apoptose at a lower level than its more resistant immortalized and tumorigenic derivatives (Pickard et al, 1995). Thus, the differences in resistance could not be explained on the basis of differences in apoptosis between the cell lines. Detailed cell cycle analysis and proliferation studies, however, showed that the normal human fibroblast cell line ceased to proliferate in response to increasing concentrations of 5-FU and entered a permanent growth arrest at a 5-FU concentration as low as $1 \times 10^{-5}$M. This was in contrast to the immortal and tumorigenic cell lines that continued to proliferate at a 5-FU concentration of $1 \times 10^{-5}$M, apparently regardless of the drug insult. This suggested that the $G_1$ checkpoint had been lost in these cell lines. The loss of p53 function may be an important factor in determining the increased resistance of the immortalized and tumorigenic cell lines to 5-FU.

### IS THERE A ROLE FOR DNA DAMAGE RESPONSE IN RESISTANCE TO MTX AND PALA?

Recent studies have identified increased levels of p53 protein that coincide with the appearance of strand breaks induced by all three of the antimetabolites MTX, PALA (Nelson and Kastan, 1994) and 5-FU (Fritsche et al, 1993). However, the evidence of a strong salvage pathway involvement in the resistance to MTX and PALA allows one to speculate that the salvage pathways may be operating to repair and synthesize DNA to such a degree that the strand breaks expected to occur in response to antimetabolite insult fail to materialize. For example, in the case of the resistance of the human fibroblast cell

© Cancer Research Campaign 1997

lines to PALA (Figure 4B) (Pickard and Kinsella 1996), all the substrates distal to the block (Figure 2) will have to be used. Moreover, the nucleosides and bases in the surrounding environment can be used by the salvage pathways to regenerate any depleted pools. Any available thymidine will be converted to TTP and any available uridine will be converted to CTP and TTP. Thus, the nucleotides required for DNA synthesis and repair continue to be provided, and so no strand breaks occur and no DNA damage response pathway is initiated. With the addition of dipyridamole and the inhibition of nucleoside and nucleobase transport, the levels of available uridine and thymidine for the salvage pathways are greatly decreased and the availability of CTP and TTP is diminished. As a result, DNA synthesis and repair are inhibited and DNA strand breaks occur, eliciting a delayed DNA damage response in the presence of normal p53 in the KMS cell line and allowing the continued proliferation of the KMST and KN-NM cell lines with their dysfunctional p53. If we look in detail at the effect of dipyridamole (5 μM) on the resistance of the three cell lines to PALA (Figure 4B), we see that dipyridamole increases the intrinsic sensitivities of both the immortalized (KMST) and the tumorigenic (KN-NM) cell lines so that they are equally sensitive and increases the sensitivity of the already sensitive normal (KMS) cell line. However, even in the presence of dipyridamole, there is still a difference in resistance to PALA between the normal and the more tumorigenic cell lines (Figure 4B). Thus, the differing abilities of the cell lines to elicit a DNA damage response explains the enhanced resistance of the KMST and KN-NM cell lines to PALA compared with the KMS cell line, even in the presence of dipyridamole (Figure 4b). There are therefore two clear components to the response of these cells to PALA.

The situation with MTX (Figure 4A) is more complicated but one can speculate that the same principles apply (Pickard and Kinsella, 1996). We know from the 5-FU data that the normal KMS cell line responds to DNA damage. Thus, the differences in the resistance of these particular human fibroblast cell lines to both MTX and PALA are clearly not only a function of differences in the salvage pathways but also of differences in the abilities of the cells to elicit a p53-dependent DNA damage response. In the case of 5-FU (Figure 4C), one has to assume in the absence of an effect of dipyridamole that the drug is mediating its effects via its direct action on DNA (Parker and Cheng, 1990) and that, as stated previously, only the DNA damage response is important. No growth arrest was observed in any of the cell lines at concentrations of PALA up to and including 10 mM, except in the case of the normal cell line in the presence of dipyridamole.

Recently, however, in a different human fibroblast cell line (Linke et al, 1996), UTP, GTP and CTP depletion following treatment with PALA (or pyrazofurin) resulted in reversible antimetabolite-induced growth arrest in the $G_1$ phase of the cell cycle, which correlated with p53 induction in the absence of apparent DNA damage. In the same fibroblast cell line, MTX and 5-FU treatment resulted in an early S phase arrest that could be prevented by co-treatment with the salvage pathway substrate thymidine. Thus, Linke et al (1996) speculated that p53 might play a role in the induction of a quiescent state in response to metabolite depletion and senescence or permanent arrest, such as that reported by the authors in human fibroblasts in response to 5-FU (Pickard et al, 1995) and by Di Leonardo et al (1994) in response to irradiation in the presence of DNA damage. It is known that p21, a downstream effector of p53, increases reversibly in quiescent cells and irreversibly in senescent cells (Noda et al, 1994). These studies and those of Li et al (1995) also provide evidence of a link between p53 and the product of retinoblastoma tumour-suppressor gene (RB) in the resistance of certain cells to the chemotherapeutic antimetabolites (Almasan et al, 1995; Li et al, 1995). RB codes for a nuclear protein implicated in the transition from the $G_1$ to S phase of the cell cycle. The $G_1$ cyclin-dependent kinase (cdk) complexes, which are inhibited by p21 a downstream effector of p53, phosphorylate the RB protein (Harper et al, 1993; Dulic et al, 1994), allowing the release of the transcriptional activator E2F and the activation of S-phase genes, such as DHFR (Blake et al, 1989; Slansky et al, 1993) and TS (Johnson, 1994). Consistent with this, growth inhibition by MTX and 5-FU was increased, and DHFR and TS activities and expression were correspondingly decreased in SaOS-2 human sarcoma cells containing a truncated RB protein that had been stably transfected with an RBcDNA (Li et al, 1995).

### CLEAR EVIDENCE FOR A ROLE FOR THE P53 DAMAGE RESPONSE IN ACQUIRED AND INHERENT DRUG RESISTANCE

The evidence for p53 being involved in both acquired and inherent resistance is consistent with what we know about the role of p53 (Lane, 1992; Levine et al, 1994). p53 plays an important role in genetic stability as a consequence of its ability to prevent the entry of damaged cells into the S phase of the cell cycle and thus preventing the replication of damaged DNA. It is known that many environmental insults and cancer therapies, including administration of the chemotherapeutic antimetabolites MTX, PALA and 5-FU, increase the levels of p53 protein expression, leading to either arrest in the $G_1$ phase of the cell cycle or apoptosis (Kastan et al, 1991; Clarke et al, 1992; Kuerbitz et al, 1992; Livingstone et al, 1992; Yin et al, 1992; Lowe et al, 1993*a,b*; Di Leonardo et al, 1994; Pickard et al, 1995). As cited above, the $G_1$ arrest pathway probably involves activation of p21, which inhibits $G_1$ cyclin–cdk complexes and which in turn inhibits phosphorylation of the RB protein and progression into the S phase of the cell cycle (Harper et al, 1993; Xiong et al, 1993; Dulic et al, 1994). It is also likely that other factors downstream of p53 are involved in this arrest response. Mutations in the *p53* gene eliminate these p53-dependent responses. Mutations in the *p53* gene are the commonest genetic change in human tumours and occur in 60% of tumours overall and 75% of colorectal malignancies (Hollstein et al, 1991; Levine et al, 1991). The presence of a mutated or dysfunctional *p53* gene product will permit the continued replication of damaged DNA, producing the conditions that facilitate gene amplification. This is consistent with the observation that cells with no p53 protein (null mutation) amplify their DNA at least a million times more readily than cells containing the normal wild-type *p53* gene product (Livingstone et al, 1992; Yin et al, 1992). Similarly, in situations of inherent resistance to single doses of chemotherapeutic antimetabolites, facilitated by the 'salvage pathways' of purine and pyrimidine biosynthesis, mutant or dysfunctional p53 allows the cells that eventually accumulate DNA damage (because of restricted substrate availability) to continue to proliferate and therefore manifest their resistance. Loss of the p53 damage response therefore contributes to both acquired and inherent drug resistance.

### CLINICAL IMPLICATIONS

Support for the circumvention of the de novo pathways of purine and pyrimidine biosynthesis by the salvage pathways comes from the observation that there are sufficient levels of nucleotides and

© Cancer Research Campaign 1997

nucleosides in human plasma to overcome the inhibitory and cytotoxic effects of the drugs (Gordon, 1985; Sinkeler et al, 1994). The plasma nucleotide concentrations represent a balance between the release of nucleotides and their degradation by extracellular nucleotidases (Boyle et al, 1989). In addition, the cytotoxic therapy itself leads to destruction of tumour cells, which is thought to provide high local concentrations of nucleosides and nucleotides in tumour patients (Fox et al, 1991).

However, few studies have dealt with the genetic factors that might control the resistance (inherent and acquired) to DNA-damaging agents and chemotherapeutic antimetabolites in vivo. The recent evidence of a role for the tumour-suppressor gene *p53* in mediating the response of cells to chemotherapeutic agents in vitro (Lowe et al, 1993*b*; Nelson and Kastan, 1994; Pickard et al, 1995) suggests that p53 dysfunction assessed quite simply by immunohistochemistry (Hall and Lane, 1994) might provide us with a preliminary predictor of the likelihood of a response to chemotherapy for individual tumours. On theoretical grounds, one can predict that the loss of normal p53 function will be associated with more rapidly advancing disease because of the fact that the replication of damaged DNA will be associated with the induction of additional point mutations and loss of heterozygosity (Harris and Hollstein, 1993; Greenblatt et al, 1994). Indeed, p53 protein expression has already been implicated as an independent prognostic indicator in carcinomas of the colon, stomach, breast, bladder and lung (NSCLC) (Dowell and Hall, 1994). The availability of immunohistochemical and polymerase chain reaction (PCR)-based diagnostic techniques means that studies can be performed on archival material, even from completed clinical trials. This will provide clinicians with the opportunity both to identify the more aggressive tumours and assess the significance of p53 and its related pathways and pathway molecules, e.g. p21, Gadd45, bcl2, bax, mdm2, p16 and RB (Di Leonardo et al, 1994; Hartwell and Kastan, 1994; Miyashita et al, 1994; Reed, 1994) in predicting chemotherapeutic and other therapeutic responses. Such studies can address the specific and general hypotheses associated with the progression to malignancy. For example, certain tumours with p53 mutations might be more resistant to drug-induced apoptosis, while others may be more sensitive to amplification of genes which might influence therapy. In the clinical situation, p53 mutations have been associated with resistance to chemotherapy in haematological malignancies (Fan et al, 1994; Hecker et al, 1994; Wattell et al, 1994) and in a study by the authors on 59 advanced colorectal carcinomas (Brett et al, 1996). Conversely, in testicular tumours elevated levels of Wtp53 may contribute to their sensitivity to DNA-damaging chemotherapeutic agents, which might explain their high cure rate (Chresta et al, 1996; Chresta and Hickman, 1996; Lutzker and Levine, 1996). Moreover, tumours lacking RB or having functional abnormalities of this protein might be more resistant to treatment with drugs that target DHFR or TS. For example, cell lines from RB patients and small-cell lung carcinomas lacking a functional RB protein are intrinsically resistant to MTX. Cancer cell survival after chemotherapy will depend on the specific cell cycle checkpoints or repair functions that have been lost. Greater susceptibility to these agents will be observed when the repair function is most important and greater resistance when it is not and the cells continue to cycle or resist apoptosis. We can predict a time when it will be possible to characterize tumours for these functions and thereby predict their responses to specific therapies in the clinic. Assessing the p53 and RB status of tumours would seem an important and worthwhile first step in the process of identifying subsets of patients with favourable and unfavourable prognoses in response to standard treatment protocols.

### ACKNOWLEDGEMENTS

ARK and MP are both funded by the North West Cancer Research Fund. The authors would like to thank Mr NA Andrews for careful reading of the manuscript.

### REFERENCES

Advanced Colorectal Cancer Meta-analysis Project (1992) Modulation of fluorouracil by leukovorin in patients with advanced colorectal cancer: Evidence in terms of response rate. *J Clin Oncol* **10**: 896–903

Allegra CJ (1990) Antifolates. In *Cancer Chemotherapy Principal and Practice* Chamber CA and Collins JA (eds), pp. 110–153 Lippincott: Philadelphia

Allegra CJ, Fine RL, Drake JC and Chabner BA (1986) The effect of methotrexate on intracellular folate pools in human MCF-7 breast cancer cells evidence for direct inhibition of purine synthesis. *J Biol Chem* **261**: 6478–6485

Allegra CJ, Hoang K, Yoh GC, Drake JC and Baram J (1987) Evidence for direct inhibition of de novo purine synthesis in human MCF-7 breast cells as a principal mode of metabolic inhibition by methotrexate. *J Biol Chem* **262**: 13520–13526

Almasan A, Linke SP, Paulson TG, Huang LC and Wahl GM (1995) Genetic instability as a consequence of inappropriate entry into and progression through S-phase. *Cancer Metastasis Rev* **14**: 59–73

Barbour KN, Berger SH and Berger FG (1990) Single amino acid substitution defines a naturally occurring genetic variant of human thymidylate synthase. *Mol Pharmacol* **37**: 515–518

Berger SH, Jenh CH and Johnston LF (1985) Thymidylate synthase overproduction and gene amplification in fluorodeoxuridine resistant human cells. *Mol Pharmacol* **28**: 461–467

Blake MC and Azizkhan JC (1989) Transcription factor E2F is required for efficient expression of hamster dihydrofolate reductase gene in vitro and in vivo. *Mol Cell Biol* **9**: 4994–5002

Bojan F, Kinsella AR and Fox M (1983) Effect of tumour promoter 12-O-tetradecanoylphorbol-13-acetate on the recovery of methotrexate-, N-phosphonacetyl-L- aspartate-and cadmium-resistant colony-forming mouse and hamster cells. *Cancer Res* **43**: 5217–5221

Borchers AH, Kennedy DA and Straw JA (1990) Inhibition of DNA excision repair by methotrexate in Chinese ovary cells following exposure to ultraviolet irradiation or ethylmethane sulfonate. *Cancer Res* **50**: 1786–1789

Boyle JM, Hey Y and Fox M (1989) Nucleotide ectoenzyme activities of human and Chinese hamster fibroblasts in tissue culture. *Biochem Genet* **27**: 655–659

Brennan J, O'Connor T, Makuch RW, Simmons AM, Russell E, Linnoila RI, Phelps RM, Gazdar AF, Inde DC and Johnson BE (1991) Myc family DNA amplification in 107 tumours and tumour cell lines from patients with small cell lung cancer treated with different combination chemotherapy regimens. *Cancer Res* **51**: 1708–1712

Brett MC, Pickard M, Green B, Howel-Evans A, Smith D, Kinsella A and Poston G (1996) p53 protein over expression and response to biomodulated 5-fluorouracil chemotherapy in patients with advanced colorectal cancer. *Eur J Surg Oncol* **22**: 182–185

Brown PC, Tlsty TD and Schimke RT (1983) Enhancement of methotrexate resistance and dihydrofolate reductase gene amplification by treatment of mouse 3T6 cells with hydroxyurea. *Mol Cell Biol* **3**: 1097–1107

Cabral S, Leis S, Bover L, Nembrot M and Mordoh J (1984) Dipyridamole inhibits reversion by thymidine of methotrexate effect and increases drug uptake in Sarcoma 180 cells. *Proc Natl Acad Sci USA* **81**: 3200–3303

Cadman E and Benz C (1980) Uridine and cytidine metabolism following inhibition of de novo pyrimidine synthesis by pyrazofurin. *Biochimica et Biophysica Act* **609**: 372–382

Camici M, Tozzi MG, Allegrini S, Delcorso A, Sanfilippo O, Daidone MG, Demarco C and Ipata PL (1990) Purine salvage enzyme activities in normal and neoplastic human tissues. *Cancer Biochem Biophys* **11**: 201–209

Chabner BA and Collins JA (1990) *Cancer Chemotherapy. Principles and Practice. Lippincott: Philadelphia*

Chan TCK and Howell SB (1985) Mechanism of synergy between N-Phosphonacetyl-L- aspatate and dipyridamole in a human ovarian carcinoma cell line. *Cancer Res* **45**: 3598–3604

© Cancer Research Campaign 1997

Cheng YC and Nakayama K (1983) Effects of 5-fluoro-2-deoxyuridine on DNA metabolism in Hela cells. *Molec Pharmacol* **23**: 171–174

Chu E, Drake JC and Koeller DM (1991) Induction of thymidylate synthase associated with multidrug resistance in human breast and colon cancer cell lines. *Mol Pharmacol* **39**: 136–143

Cillo C, Ling C and Hill RP (1989) Drug resistance in KHT fibrosarcoma cell lines with different metastatic ability. *Int J Cancer* **43**: 107–111

Clarke AR, Purdie CA, Harrison DJ, Morris RG, Bird CC, Hooper ML and Wyllie AH (1993) Thymocyte apoptosis induced by p53-dependent and independent pathways. *Nature* **362**: 849–852

Collins KD and Stark GR (1971) Aspartate transcarbamylase interaction with the transition state analogue N-(phosphonacetyl)-L-aspartate. *J Biol Chem* **246**: 6599–6605

Chresta CM and Hickman JA (1996) Oddball p53 in testicular tumours. *Nature Med* **2**: 745–746

Chresta CM, Masters JRW and Hickman JA (1996) Hypersensitivity of human testicular tumours to etoposide-induced apoptosis associated with functional p53 and a high Bax. Bcl2 ratio. *Cancer Res* **56**: 1834–1841

Di Leonardo A, Linke SP, Clarkin K and Wahl GM (1994) DNA damage triggers a prolonged p53-dependent G7-arrest and long-term induction of CiPl in normal human fibroblasts. *Genes Devel* **8**: 2540–2551

Dive C and Hickman JA (1991) Drug target interactions: only the first step in the commitment to a programmed cell death. *Br J Cancer* **64**: 192–196

Dowell SP and Hall PA (1994) The clinical relevance of the p53 tumour suppresser gene. *Cytopathology* **5**: 133–145

Dulic V, Kaufmann WK, Wilson SJ, Tlsty TD, Lees E, Harper JW, Elledge SJ and Reed SI (1994) p53-dependent inhibition of cyclin-dependent kinase activities in human fibroblasts during radiation-induced G1 arrest. *Cell* **76**: 1013–1023

Erilichman C (1992) Pharmacology of anticancer drugs. In *The Basic Science of Oncology* Tannock IF and Hill RP. (eds) McGraw Hill

Evans RM, Laskin JD and Hakala MT (1980) Assessment of growth limiting events caused by 5-fluorouracil in mouse cells and in human cells. *Cancer Res* **40**: 4113–4122

Fan S, El-Deiry WS, Bae I, Freeman J, Jondle D, Bhatia K, Fornace AJ, Magrath I, Kohn KW and O'Connor PM (1994) p53 mutations are associated with decreased sensitivity of human lymphanic cells to DNA-damaging agents. *Cancer Res* **54**: 5824–5830

Flintoff WE, Davidson SV and Siminovitch L (1976) Isolation and partial characterisation of three methotrexate-resistant phenotypes from Chinese hamster ovary cells. *Somat Cell Gen* **2**: 245–261

Fox M, Boyle JM and Kinsella AR (1991) Nucleoside salvage and resistance to antimetabolite anticancer agents. *Br J Cancer* **64**: 428–436

Fritsche M, Haessler C and Brandner G (1993) Induction of nuclear accumulation of the tumour suppressor protein p53 by DNA damaging agents. *Oncogene* **8**: 307–318

Giulotto E, Knights C and Stark GR (1987) Hamster cells with increased rates of DNA amplification, a new phenotype. *Cell* **48**: 837–845

Gordon JL (1985) Extracellular ATP, effect, sources and fate. *Biochem J* **233**: 309

Goz B and Jeffs L (1994) The enhancement of the frequency of resistance to N-Phosphonacetyl-L-aspartate and methotrexate by 1-B-D-arabinofuranosylcytosine. The effect of dipyridamole. *J Pharmacol Exp Ther* **270**: 480–484

Goz B, Carl PL and Tlsty TD (1989) 1-B-D Arabino-furanosylcytosine enhancement of resistance to several antineoplastic drugs in mammalian tissue culture cells. *Mol Pharmacol* **36**: 360–365

Greenblatt MS, Bennett WP, Hollstein M and Harris CC (1994) Mutations in the p53 tumour suppressor gene: clues to cancer etiology and molecular pathogenesis. *Cancer Res* **54**: 4855–4878

Grem JL (1992) Biochemical modulation of fluorouracil by dipyridamole: preclinical and clinical experience. *Semin Oncol* **19**: 56–65

Grem JL and Fischer PH (1985) Augmentation of 5-fluorouracil cytotoxicity in human colon cancer cells by dipyridamole. *Cancer Res* **45**: 2967–2972

Grem JL and Fischer PH (1986) Alteration of flurouracil metabolism in human colon cancer cells by dipyridamole with a selective increase in fluorodeoxyuridine monophosphate levels. *Cancer Res* **46**: 6191–6199

Grem JL and Fischer PH (1989) Enhancement of 5-fluorouracil's anticancer activity by dipyridamole. *Pharmac Ther* **40**: 349–371

Hall PA and Lane DP (1994) p53 in tumour pathology: can we trust immunohistochemistry? –revisited. *J Pathol* **172**: 1–4

Harper JW, Adami GR, Wei N, Kegomarsi K and Elledge SJ (1993) The p21 Cdk-interacting protein Cipl is a potent inhibitor of G1 cyclin-dependent kinases. *Cell* **75**: 805–816

Harris CC and Hollstein M (1993) Clinical implications of the p53 tumour suppressor gene. *New Eng J Med* **329**: 1318–1327

Hartwell LH and Kastan MB (1994) Cell cycle control and cancer. *Science* **266**: 1821–1828

Hecker S, Sauerbrey A and Volm M (1994) p53 expression and poor prognosis in childhood acute lymphoblastic leukaemia. *Anticancer Res* **14**: 2759–2761

Heidelberger C, Chauduri NK, Danenberg P et al (1957) Fluorinated pyrimidines a new class of tumour inhibitory compound. *Nature* **179**: 663–666

Heidelberger C, Danenberg PV and Moran RG (1983) Fluorinated pyrimidines and their nucleosides. *Adv Enzymol* **54**: 57–119

Hollstein M, Sidransky D, Vogelstein B and Harris CC (1991) p53 mutations in human cancers. *Science* **253**: 49–53

Houghton JA, Maroda SJ Jnr, Philips JO and Houghton PJ (1981) Biochemical determinants of responsiveness to 5-fluorouracil and its deviates in xenografts of human colorectal adenocarcinomas in mice. *Cancer Res* **41**: 144–149

Huang L-C, Clarkin KC and Wahl GM (1996) Sensitivity and selectivity of DNA damage sensor responsible for activating p53-dependent G1 arrest. *Proc Natl Acad Sci USA* **93**: 4827–4832

Ishizaka Y, Chernov MV, Burns CM and Stark GR (1995) p53-dependent growth arrest of REF 52 cells containing newly amplified DNA. *Proc Natl Acad Sci USA* **92**: 3224–3228

Jastreboff MM, Kedzherska B and Rode W (1983) Altered thymidylate synthase in fluoro-deoxyuridine-resistant cultured hepatoma cells. *Biochem Pharmacol* **32**: 2259–2267

Johnson LF (1994) Postranscriptional regulation of thymidylate synthase gene expression. *J Cell Biochem* **54**: 387–392

Kastan MB, Onyekwere O, Sidransky D, Vogelstein B and Craig RW (1991) Participation of p53 protein in the cellular response to DNA damage. *Cancer Res* **51**: 6304–6311

Kennedy DG, Van Den Berg HW, Clarke R and Murphy RF (1986) Enhancement of methotrexate cytotoxicity towards the MDA. MB. 436 human breast cancer cell line by dipyridamole. *Biochem Pharmacol* **35**: 3053–3056

Kuerbitz SJ, Plunkett BS, Walsh WV and Kastan MB (1992) Wild-type p53 in a cell cycle checkpoint determinant following invadiation. *Proc Natl Acad Sci USA* **89**: 7491–7495

Kinsella AR and Haran MS (1991) Decreasing sensitivity to cytotoxic agents parallels increasing tumorigenicity in human fibroblasts. *Cancer Res* **51**: 1855–1859

Lane DP (1992) p53 guardian of the genome. *Nature* **358**: 15–16

Levine AJ, Momand J and Finlay CA (1991) The p53 tumour suppressor gene. *Nature* **351**: 453–456

Levine AJ, Perry A, Chang A, Silver A, Dittmer D, Wu M and Welsh D (1994) The 1993 Walter Hubert Lecture: The role of the p53 tumour-suppressor gene in tumorigenesis. *Br J Cancer* **69**: 409–416

Li WW, Fan J, Hochhauser D, Banerjee D, Zielinski Z, Almasan A, Yin YX, Kelly R, Wahl GM and Bertino JR (1995) Lack of a functional retinoblastoma protein mediates increased resistance to antimetabolites in human sarcoma cells. *Proc Natl Acad Sci USA* **92**: 10436–10440

Linke SP, Clarkin KC, Di Leonardo A, Tsou A and Wahl GM (1996) A reversible, p53-dependent G0/G1 cell cycle arrest induced by ribonucleotide depletion in the absence of detectable DNA damage. *Genes Devel* **10**: 934–947

Livingstone LR, White A, Sprouse J, Livanos E, Jacks T and Tlsty TD (1992) Altered cell cycle arrest and gene amplification potential accompany loss of wild-type P53. *Cell* **70**: 923–935

Lorico A, Toffoli G, Boiocchi M, Erba E, Broggini M, Rappa G and Dincalci M (1988) Accumulation of DNA strand breaks in cells exposed to Methotrexate or N10-propargyl-5, 8-dideazafolic acid. *Cancer Res* **48** 2036–2041

Lowe S, Schmitt EM, Smith SW, Osborne BA and Jacks T (1993*a*) p53 is required for radiation-induced apoptosis in mouse thymocytes. *Nature* **362**: 847–849

Lowe SW, Ruley EH, Jacks T and Housman DE (1993*b*) p53-dependent apoptosis modulates the cytotoxicity of anti-cancer agents. *Cell* **74**: 957–967

Lucke-Huhle C (1989) Review: Gene amplification – a cellular response to genotoxic stress. *Mol Toxicol* **2**: 237–253

Lucke-Huhle C (1994) Permissivity for methotrexate-induced DHFR gene amplification correlates with the metastatic potential of rat adenocarcinoma cells. *Carcinogenesis* **15**: 695–700

Lutzker SG and Levine AJ (1996) A functionally inactive p53 protein in teratocarcinoma cells is activated by either DNA damage or cellular differentiation. *Nature Med* **2**: 804–809

Machover D, Schwarzenberg L, Goldschmidt E et al (1982) Treatment of advanced colorectal and gastric adenocarcinoma with 5-FU combined with high-dose folinic acid: a pilot study. *Cancer Treat Rep* **66**: 1803–1807

Madoc-Jones H and Bruce WR (1968) On the mechanism of the lethal action of 5-fluorouracil on mouse L cells. *Cancer Res* **28**: 1976–1981

© Cancer Research Campaign 1997

Mandel G (1969) The incorporation of 5-fluorouracil into RNA and its molecular consequences. In *Progress in Molecular and Subcellular Biology*, Hahn FE (ed.), pp. 82–135. Springer: New York

Martin DS, Stozfi RL, Sawyer RC, Spiegelman S, Casper ES and Young CW (1983) Therapeutic utility of utilising low doses of N-(phosphonacetyl)-L- aspartate in combination with 5-fluorouracil. A murine study with clinical relevance. *Cancer Res* **43**: 2317–2321

Marz R, Wohlhueter RM and Plagemann PGW (1977) Growth rate of cultured Novikoff rat hepatoma cella as a function of the rate of thymidine and hypoxanthine transport. *J Membr Biol* **34**: 277–288

McIlwrath AJ, Vasey PA, Ross GM and Brown R (1994) Cell cycle arrests and radiosensitivity of human tumor cell lines: dependence on WT p53 for radiosensitivity. *Cancer Res* **54**: 3718–3722

Miller EM, Willson JF and Fischer PH (1987) Folinic acid alters the mechanism by which dipyridamole increases the toxicity of fluorouracil in human colon cancer cells. *Proc Am Assoc Cancer Res* **28**: 326

Miyashita T, Krajewski S, Krajewska M, Wang HG, Lin HK, Liebermann DA, Hofmann B and Reed JC (1994) Tumour suppressor p53 is a regulator of bcl-2 and bax gene expression in vitro and in vivo. *Oncogene* **9**: 1799–1805

Moyer JD, Smith PA, Levy EJ and Handschumacher RE (1982) Kinetics of N-(phosphonacetyl)-L-aspartate and pyrozafurin depletion of pyrimidine ribonucleotide and deoxynucleotide pools and their nucleic acid synthesis in intact and permebilised cells. *Cancer Res* **42**: 4525–4531

Natsumeda Y, Ikegami T, Olah E and Weber G (1989) Significance of purine salvage in circumventing the action of antimetabolites in rat hepatoma cells. *Cancer Res* **49**: 88–92

Nelson JA and Drake S (1984) Potentiation of methotrexate toxicity by dipyridamole. *Cancer Res* **44**: 2493–2496

Nelson WG and Kastan MB (1994) DNA strand breaks: the DNA template alterations that trigger P53-dependent DNA damage response pathways. *Mol Cell Biol* **14**: 1815–1823

Noda A, Ning Y, Venable SF, Pereira-Smith OM and Smith JR (1994) Cloning of senescent cell-derived inhibitors of DNA synthesis using and expression screen. *Exp Cell Res* **211**: 90–98

Otto E, Mccord S and Tlsty TD (1989) Increased incidence of CAD gene amplification in tumorigenic rat lines as an indicator of genomic instability. *J Biol Chem* **264**: 3390–3396

Parker WD and Cheng YC (1990) Metabolism and mechanism of 5-fluorouracil. *Pharmac Ther* **4**: 381–395

Perry ME, Commane M and Stark GR (1992) Simian virus 40 large tumour antigen alone or two co-operating oncogenes convert REF 52 cells to a state permissive for gene amplification. *Proc Natl Acad Sci USA* **89**: 8112–8116

Plagemann PGW, Marz R and Wohlhueter RM (1978) Transport and metabolism of deoxycytidine and I-B-D-arabinofuranosylcytosine into cultured Novikoff rat hepatoma cells, relationship to phosphorylation and regulation of triphosphate synthesis. *Cancer Res* **38**: 978–989

Pickard M and Kinsella AR (1996) Influence of both salvage and DNA damage response pathways with resistance to chemotherapeutic antimetabolites. *Biochem Pharmacol* **52**: 425–431

Pickard M, Dive C and Kinsella AR (1996) Differences in resistance to 5-FU as a function of cell cycle delay and not apoptosis. *Br J Cancer* **72**: 1389–1396

Reed JC (1994) Bcl-2 and the regulation of programmed cell death. *Cell Biol* **124**: 1–6

Rice GC, Hoy C and Schimke RT (1986) Transient hypoxia enhances the frequency of dihydrofolate reductase gene amplification in Chinese hamster ovary cells. *Proc Natl Acad Sci USA* **83**: 5978–5982

Schimke RT (1984*a*) Gene amplification, drug resistance and cancer. *Cancer Res* **44**: 1735–1741

Schimke RT (1984*b*) Gene amplification in cultured animal cells. *Cell* **37**: 705–713

Schimke RT, Sherwood SW, Hill AB and Johnston RN (1986) Over replication and recombination in higher eukaryotes potential consequences and biological implications. *Proc Natl Acad Sci USA* **83**: 2157–2161

Schwab M and Amler LC (1990) Amplification of cellular oncogenes. A predictor of clinical outcome in human cancer. *Genes Chromosomes Cancer* **1**: 181–193

Schwartz J, Alberts D, Einspahr J, Peng YM and Spears P (1987) Dipyridamole potentiation of FUDR activity against human colon cancer in vitro and in patients. *Proc Am Soc Chem Oncol* **6**: 83

Sinkeler S, Joesten E, Weaver R, Binkhorst R and Oel L (1986) Skeletal muscle adenosine, inosine and hypoxanthine release following ischaemic forearm exercise in myoadenylate deaminase deficiency and McArdle's disease. *Adv Exp Med Biol* **195B**: 517

Slansky JE, Kaelin WG and Farnham PJ (1993) A protein synthesis-dependent increase in E2F1mRNA correlates with growth regulation of the dihydrofolate reductase promoter. *Mol Cell Biol* **13**: 1610–1618

Sommer A and Santi DV (1974) Purification and amino acid analysis of an active site peptide from thymidylate synthetase containing covalenty bound 5′-fluoro-2′-deoxyuridylate and methylene tetrachloride. *Biochem Biophys Res Commun* **57**: 689–696

Sotos GA, Grogan L and Allegra CJ (1994) Preclinical and clinical aspects of biomodulation of 5-fluorouracil. *Cancer Treatment Rev* **20**: 11–49

Stark GR (1986) DNA – amplification in drug resistant cells and in tumours. *Cancer Surv* **5**: 1–23

Stark GR (1993) Regulation and mechanisms of gene amplification. *Adv Cancer Res* **61**: 87–114

Stark GR and Wahl GM (1984) Gene amplification. *Ann Rev Biochem* **53**: 447–491

Stark GR, Debatisse M, Giulotto E and Wahl GM (1989) Recent progress in understanding mechanisms of mammalian gene amplification. *Cell* **57**: 901–908

Swyryd EA, Seaver SS and Stark GR (1974) N-(Phosphonacetyl)-L-aspartate transcarbmylase blocks proliferation of mammalian cells in culture. *J Biol Chem* **249**: 6945–6950

Szallasi A, Fox M and Kinsella AR (1988) TPA-enhancement of the recovery of methotrexate and N-(phosphonacetyl)-L-aspartate-resistant mouse 3T6 cell clones is associated with transient alteration of cell cycle progression. *Int J Cancer* **42**: 84–86

Tlsty TD (1990) Normal diploid human and rodent cells lack a detectable frequency of gene amplification. *Proc Natl Acad Sci* **87**: 3132–3136

Tlsty TD, Brown PC, Johnston R and Schimke RT (1982) Enhanced frequency of generation of methotrexate resistance and dihydrofolate gene amplification in cultured mouse and hamster lines. In *Gene Amplification*, Schimke RT (ed.), pp. 231–238 , Cold Spring Harbor Laboratory: Cold Spring Harbor, New York

Tlsty TD, Brown PC and Schimke RT (1984) UV radiation facilitates methotrexate resistance and dihydrofolate reductase gene amplification in cultured murine cells. *Mol Cell Biol* **4**: 1050–1056

Tlsty TD, Margolin BH and Lum K (1989)Differences in the rates of gene amplification in non-tumorigenic and tumorigenic cell lines as measured by Luria–Deltrück fluctuationanalysis. *Proc Natl Acad Sci* **86**: 9441–9445

Tsvaris N, Zinelis A, Karvounis N, Beldecos D, Mylonacis N, Zamanis N, Bacoyannis C, Valilis P, Antonopoulos A and Kosmidis P (1990) Multimodal biochemical modulation of 5-fluorouracil activity in advanced colorectal cancer with allopurinol, folinic acid and dipyridamol. *J Chemother* **2**: 123–126

Ullman B, Lee M, Martin DW and Santi DV (1978) Cytotoxicity of 5-fluoro-2-deoxyuridine: requirement for reduced folate cofactors and antagonism of methotrexate. *Proc Natl Acad Sci USA* **75**: 980–983

Vogel SJ, Presant CA and Ratkin GA (1979) Phase 1 study of thymidine plus 5-fluorouracil infusions in advanced colorectal carcinoma. *Cancer Treat Rep* **63**: 1–5

Van Mouwerik TJ, Pangallo CA, Willson JKV and Fischer PH (1987) Augmentation of methotrexate cytotoxicity in human colon cancer cells achieved through inhibition of thymidine salvage by dipyridamole. *Biochem Pharmacol* **36**: 809–814

Wahl GM, Padgett RA and Stark GR (1979) Gene amplification causes overproduction of the first three enzymes of UMP synthesis in N-(Phosphonacetyl)-L-aspartate-resistant hamster cells. *J Biol Chem* **254**: 8679–8689

Wattell E, Preudhomme C, Hecquet B, Vanrumbeke M, Quesnel B, Dervite I, Morel P and Fanaux (1994) P53 mutations are associated with resistance to chemotherapy and short survival in haematologic malignancies. *Blood* **84**: 3148–3157

Waxman S, Bruckner H, Wagle A and Schreiber C (1978) Potentiation of 5-fluorouracil (5FU). Antimetabolic effect by leukovorin (LV). *Proc Am Assoc Cancer Res* **19**: 149

Weber G (1993) Biochemical strategy of cancer cells and the design of chemotherapy: GHA Clowes Memorial Lecture. *Cancer Res* **43**: 3466–3492

Weber G and Prajda N (1994) Targeted and non-targeted actions of anti-cancer drugs. *Adv Enzyme Regul* **34**: 71–89

Windle BE and Wahl GM (1992) Molecular dissection of mammalian gene amplification: new mechanistic insights revealed by analyses of very early events. *Mutat Res* **276**:199–224

Wright JA, Smith HS, Watt FM, Hancock MC, Hudson DL and Stark GR (1990) DNA amplification is rare in normal human cells. *Proc Natl Acad Sci USA* **87**: 1791–1795

© Cancer Research Campaign 1997

Xiong Y, Hannon H, Zhang H, Casso D, Kobayashi R and Beach D (1993) p21 is a universal inhibitor of cyclin kinases. *Nature* **366**: 701–704

Yin Y, Tainsky MA, Bischoff FZ, Strong LC and Wahl GM (1992) Wild type P53 restores cell cycle control and inhibits gene amplification in cells with mutant P53 alleles. *Cell* **70**: 937–948

Yoshia T, Stark GR and Hoogenraad NJ (1974) Inhibition by N-(phosphonacetyl)-L-asparate of aspartate transcarbamylase activity and drug-induced cell proliferation in mice. *J Biol Chem* **249**: 6951–6955

Yoshioka A, Tanaka S, Hiraoka O, Koyama Y, Hirota Y, Ayusawa D, Seno T, Garrett C and Wataya Y (1987) Deoxyribonucleoside triphosphate imbalance. 5-fluorodeoxyuridine-induced DNA double strand breaks in mouse. FM3A cells and the mechanism of cell death. *J Biol Chem* **262**: 8235–8241

© Cancer Research Campaign 1997